# Differentiation of human colon cancer cells changes the expression of **β**-tubulin isotypes and MAPs

**DOI:** 10.1038/sj.bjc.6690481

**Published:** 1999-06

**Authors:** G Carles, D Braguer, C Dumontet, V Bourgarel, A Gonçalves, M Sarrazin, J B Rognoni, C Briand

**Affiliations:** UPRES-A CNRS 6032, University of ‘la Méditerranée’, 27 Bd Jean Moulin, Marseille, 13005 Cedex 5, France; Inserm-UU53, Faculty of Medicine, 8 Av Rockefeller, Lyon, 69008, France

**Keywords:** anti-microtubule agents, taxoids, β-tubulin isotypes, microtubule-associated proteins, cell polarity

## Abstract

The human colon adenocarcinoma HT29-D4 cell line is an interesting model for studies on epithelial cell differentiation. Undifferentiated cells are malignant proliferating cells, whereas differentiated cells act like epithelial polarized cells. In the present study, we first characterized the action of taxoids on the microtubular network of HT29-D4 cells according to the state of differentiation. Microtubular bundles were found in undifferentiated cells but not in differentiated cells, even with 500-fold higher taxoid concentrations for 96 h. This finding led us to study changes in microtubules according to the polarity status of the cell. E-MAP-115 was expressed only in differentiated cells; expression of β-tubulin isotypes was altered in them relative to undifferentiated cells. Classes I, II, III, IVa and IVb isotypes were expressed in both phenotypes; however, differentiated epithelial cells displayed a specific increase in class III β-tubulin. Thus, the increase in expression of this β-tubulin isotype in differentiated cells is not restricted to neuronal cells. Moreover, these expression changes may reflect a higher stability of microtubular network in differentiated cells, which may explain the lower activity of anti-microtubule agents, independently of the mitotic process. These results indicate that the composition of microtubules should be considered as one of the criteria involved in the response of tumour cells to chemotherapy with anti-microtubule agents. © 1999 Cancer Research Campaign


*British Journal of Cancer* (1999) **80**(8), 1162–1168
© 1999 Cancer Research Campaign
Article no. bjoc.1998.0481

### Differentiation of human colon cancer cells changes the expression of β-tubulin isotypes and MAPs

**G Carles[1], D Braguer[1], C Dumontet[2], V Bourgarel[1], A Gonçalves[1], M Sarrazin[1], JB Rognoni[1] and C Briand[1]**

[1]UPRES-A CNRS 6032, University of 'la Méditerranée', Faculty of Pharmacy, 27 Bd Jean Moulin, 13005 Marseille Cedex 5, France; [2]Inserm-UU53, Faculty of Medicine, Laboratory of Analytical Cytology, 8 Av Rockefeller, 69008 Lyon, France

**Summary** The human colon adenocarcinoma HT29-D4 cell line is an interesting model for studies on epithelial cell differentiation. Undifferentiated cells are malignant proliferating cells, whereas differentiated cells act like epithelial polarized cells. In the present study, we first characterized the action of taxoids on the microtubular network of HT29-D4 cells according to the state of differentiation. Microtubular bundles were found in undifferentiated cells but not in differentiated cells, even with 500-fold higher taxoid concentrations for 96 h. This finding led us to study changes in microtubules according to the polarity status of the cell. E-MAP-115 was expressed only in differentiated cells; expression of β-tubulin isotypes was altered in them relative to undifferentiated cells. Classes I, II, III, IVa and IVb isotypes were expressed in both phenotypes; however, differentiated epithelial cells displayed a specific increase in class III β-tubulin. Thus, the increase in expression of this β-tubulin isotype in differentiated cells is not restricted to neuronal cells. Moreover, these expression changes may reflect a higher stability of microtubular network in differentiated cells, which may explain the lower activity of anti-microtubule agents, independently of the mitotic process. These results indicate that the composition of microtubules should be considered as one of the criteria involved in the response of tumour cells to chemotherapy with anti-microtubule agents.

**Keywords:** anti-microtubule agents; taxoids; β-tubulin isotypes; microtubule-associated proteins; cell polarity

Anti-microtubule agents bind to tubulin or microtubules and inhibit cell replication by acting on the mitotic spindle (Wilson and Jordan, 1995). Vinca alkaloids depolymerize microtubules; taxoids induce the formation of stable, non-functional microtubular bundles in interphase cells. These agents are among the most effective anticancer drugs, in particular the taxoids paclitaxel (Taxol®) and docetaxel (Taxotere®) for treatment of advanced ovarian and breast carcinomas. They also show activity against other carcinomas (Rowinsky et al, 1992; Dieras et al, 1996; McGuire et al, 1996). Several promising antimitotic compounds have been characterized, particularly discodermolide and epothilones, which are taxol-like compounds, and cryptophycins and dolastatins, which are depolymerizing agents (De Arruda et al, 1995; Jordan and Wilson, 1998). Moreover, analogues of paclitaxel and docetaxel display potential clinical importance. Clinical evaluation is in progress for some of these novel compounds. However, drug resistance hinders the successful use of anticancer drugs. The best understood mechanism of resistance to cytotoxic drugs including anti-microtubule agents is the MDR phenotype mediated by the multidrug transporter P-glycoprotein (P-gp) (Horwitz et al, 1993). Other possible mechanisms of specific resistance to anti-microtubule agents concern alterations in microtubule composition or dynamics. Alterations in α- and β-tubulin subunits have been described in both human and non-human paclitaxel-resistant cell lines (Schibler and Cabral, 1986; Ranganathan et al, 1998). However, mutations or changes in the expression of tubulin subunits in human tumour cells are poorly defined. Furthermore, the dynamics of microtubules assembled from purified β-tubulin isotypes differs in vitro, which influences the sensitivity to anti-microtubule drugs (Derry et al, 1997).

*Received 21 September 1998*
*Revised 9 December 1998*
*Accepted 9 December 1998*

*Correspondence to:* D Braguer

Human adenocarcinoma is often composed of both undifferentiated and differentiated cells. The differentiation status is one of the major prognostic factors. The undifferentiated cells proliferate and are highly sensitive to anticancer drugs; on the contrary, differentiated cells are classically less sensitive to chemotherapy, even if they display a less aggressive behaviour because of their low rate of proliferation. For these reasons, the human colon adenocarcinoma HT29-D4 cell line is particularly suitable to study the microtubule changes according to the phenotype of cell differentiation. Indeed, HT29-D4 cells grown with glucose proliferate and are undifferentiated; with galactose, they differentiate into an enterocyte-like polarized cell (Fantini et al, 1986; Rognoni et al, 1998).

Stability of microtubules has been implicated in the establishment of polarity in neuronal cells and fibroblasts (Baas and Black, 1990; Gurland and Gundersen, 1995). Few studies were conducted on polarized epithelial cell models such as MDCK and Caco-2 (Simons and Wandinger-Ness, 1990). Microtubules in polarized MDCK cells were more resistant to depolymerization than microtubules in non-polarized cells (Wadsworth and Bottaro, 1996). Stable microtubules often contain post-translationally modified tubulin, but acetylation and detyrosination are the result rather than the cause of microtubule stability (Bulinski and Gundersen, 1991). Other factors, such as mitogen-activated proteins (MAPs) and tubulin isotypes, are also involved in the events of microtubule stabilization (Matus, 1988; Falconer et al, 1992). Dynamic instability is cell type-specific (Cassimeris, 1993; Shelden and Wadsworth, 1993). Moreover, the molecular mechanisms responsible for the control of microtubule dynamic behaviour during cell growth and differentiation are largely unknown.

Since taxoids induced formation of microtubular bundles only in undifferentiated cells, we sought to investigate the changes in microtubules linked with the polarity status of the cells. Beside detyrosination of α-tubulin, differentiated cells displayed expression of E-MAP-115 and a specific increase in class III β-tubulin isotype. These variations may be involved in the difference of response to taxoids on HT29-D4 cells according to this differentiation state, independently of mitotic process.

### MATERIALS AND METHODS

### Cell culture and differentiation

Because of cellular heterogeneity of the original HT29 cell line, the HT29-D4 clone was isolated by limiting dilution technique. Undifferentiated HT29-D4 cells (HT29-D4 *Glu*) were routinely grown in Dulbecco's modified Eagle's medium (DMEM) (Biowhittaker, Vervier, Belgium) containing 25 mM glucose (*Glu* medium). To induce the differentiation of HT29-D4 cells, glucose was replaced by galactose (*Gal* medium). The detailed conditions of HT29-D4 cell culture were published elsewhere (Fantini et al, 1986, 1989). The doubling time was 20 ± 2 h in *Glu* medium. Differentiated HT29-D4 *Gal* cells were limited to two subcultures.

### Drugs

Paclitaxel was obtained from Sigma (St Louis, MO, USA). Docetaxel was a gift from Rhône Poulenc Rorer (Paris, France). Stock solutions of drugs (0.01 M) were made in dimethyl sulphoxide (DMSO) and were stored at –20°C. The working solutions were diluted in culture medium; the highest concentration of DMSO was 0.2% (v/v). [$^3$H]paclitaxel and [$^{14}$C]docetaxel were from Moravek Biochemicals Inc. (Brea, CA, USA) and Commissariat à l'Energie Atomique (Saclay, France) respectively.

### Transmission electron microscopy

Cells were fixed in situ with 2.5% glutaraldehyde in 0.1 M sodium phosphate buffer pH 7.3 for 1 h, washed in the same buffer with 6.84% saccharose, post-fixed in 1% osmium tetroxide, dehydrated in ethanol and embedded in Epon. Sections were observed with a Jeol 100 C electron microscope.

### Carcinoembryonic antigen measurement

The release of carcinoembryonic antigen (CEA) into the cell culture medium was determined with a commercially available CEA–EIA kit (Abbot Laboratories, Chicago, IL, USA).

### Immunofluorescence microscopy

Cells grown on glass coverslips were rinsed three times with phosphate-buffered saline (PBS), lysed with 0.5% Triton X-100, fixed with 3.7% formaldehyde, and stained with anti-α-tubulin and anti-mouse immunoglobulin, fluorescein-linked whole antibody (Ab) (Amersham, UK) as previously described (Garcia et al, 1994). For staining with anti-CEA (Biosys, Compiegne, France) and anti-β1 integrin (Immunotech-Coulter, Marseille, France), cells were blocked, incubated for 1 h at 4°C with specific Ab and fixed with 3% paraformaldehyde. They were stained with a second anti-mouse immunoglobulin Ab coupled to fluorescein and mounted on glass slides in a solution containing 90% of glycerol and 10% of PBS.

**Table 1** Intracellular[$^3$H] paclitaxel and [$^{14}$C]docetaxel in HT29-D4 *Glu* and *Gal* cells. Each point shows the mean of three independent experiments (± s.d.)

| | Drug concentration (nM) | Intracellular drug (pM mg$^{-1}$ protein) HT29-D4 *Glu* cells | HT29-D4 *Gal* cells |
|---|---|---|---|
| [$^3$H] paclitaxel | 50 | 118 ± 9 | 144 ± 7 |
| | 100 | 204 ± 11 | 265 ± 6 |
| | 500 | 397 ± 106 | 644 ± 37 |
| | 1000 | 561 ± 170 | 959 ± 44 |
| | 2000 | 1010 ± 180 | 1635 ± 92 |
| [$^{14}$C] docetaxel | 50 | 15 ± 3 | 37 ± 1 |
| | 100 | 45 ± 1 | 122 ± 7 |
| | 500 | 118 ± 1 | 249 ± 14 |
| | 1000 | 147 ± 17 | 330 ± 28 |
| | 2000 | 196 ± 3 | 491 ± 70 |

### Cell drug accumulation

Total binding of [$^3$H]paclitaxel or [$^{14}$C]docetaxel to cells was determined according to the technique of Manfredi and Horwitz (1984). Protein determination and radioactivity were measured as previously described (Garcia et al, 1994).

### Evaluation of total and cytoskeletal tubulin protein

For quantification of total tubulin protein, cells were washed twice in cold PBS and lysed at 90°C in lysis buffer containing Tris–HCl pH 6.80, 2.5% of sodium dodecyl sulphate (SDS), 5% of mercaptoethanol, and 10% of glycerol. For quantification of cytoskeletal tubulin protein, cells were washed twice in PEM/PEG buffer containing 100 mM Pipes, 1 mM ethyleneglycol-*bis*-(β aminoethyl ether) *N,N,N′,N′*-tetraacetic acid (EGTA), 1 mM magnesium chloride and 4% of polyethylene glycol (PEG) 8000. Soluble proteins were extracted for 90 s in PEM/PEG buffer containing 0.5% of Triton X-100 and $10^{-3}$ M GTP and lysed in the lysis buffer. All buffers contained protease inhibitors 1 mM phenylmethylsulphonyl fluoride (PMSF), 50 µg ml$^{-1}$ leupeptin, 1 µg ml$^{-1}$ pepstatin, 1 mg ml$^{-1}$ trypsin inhibitor and 20 µg ml$^{-1}$ aprotinin (Sigma, St Louis, MO, USA). Bio-Rad protein assay dye reagent was used to determine the protein concentration of lysates.

### Western blotting

Equal amounts of total or cytoskeletal proteins were prepared in SDS, boiled and then applied to a 10% acrylamide-SDS running gel. Proteins were blotted onto Hybond-C extra nitrocellulose membrane (Amersham, UK) with an Electrophoretic Transfer Cell apparatus (Bio-Rad Laboratories, CA, USA). Equal loadings and efficient transfer of proteins were confirmed by staining the membrane with ponceau S red before immunodetection. The membrane was blocked for 3 h with PBS containing 5% dried non-fat milk powder and 0.1% Tween-20 and then incubated overnight at 4°C with primary Abs. The membrane was incubated for 1 h in

© 1999 Cancer Research Campaign

**Table 2** Expression ratios of total and polymerized α- and β-tubulin, and post-translational modified α-tubulin in HT29-D4 *Glu* and HT29-D4 *Gal* cells

| | Ratio *Glu*/*Gal*[a] | |
|---|---|---|
| | **Total tubulin** | **Polymerized tubulin** |
| α-tubulin | 1.6 ± 0.3 | 1.1 ± 0.1 |
| Tyrosinated α-tubulin | 2.4 ± 0.5 | 1.1 ± 0.2 |
| Detyrosinated α-tubulin | – | 0.5 ± 0.1 |
| Acetylated α-tubulin | 1.6 ± 0.2 | 1.2 ± 0.3 |
| β-tubulin | 1.6 ± 0.2 | 0.8 ± 0.2 |

[a]Ratios are expressed as the densitometric value of protein in HT29-D4 *Glu* cells divided by the densitometric value of protein in HT29-D4 *Gal* cells.

secondary specific Ab conjugated with peroxydase (Amersham), washed, and processed in enhanced chemiluminescence (ECL) reagent (Amersham). Densitometric quantification was performed using TRAITIMA, a homemade software. Western blots were obtained from at least three independent experiments using separate cultures.

The primary antibodies for immunoblotting, mouse monoclonal antibody (mAb) anti-E-MAP-115 and rabbit anti-detyrosinated tubulin (*Glu* Tubulin), were generously provided by Masson and Kreis respectively; rabbit polyclonal Ab to MAP4 by Bulinski and mouse mAb to class III β-tubulin isotype by Frankfurter. Anti-α-tubulin Ab (N356) was from Amersham, anti-β-tubulin (T4026), anti-α-tyrosinated (T9028) and anti-α-acetylated (T6793) Abs from Sigma, and anti-class IV β-tubulin isotype (MU 178-UC) from Biogenex (San Ramon, CA, USA). Secondary anti-mouse (NA931) and anti-rabbit (NA934) immunoglobulins, horseradish peroxidase were from Amersham.

### RNA isolation and RT-PCR analysis

Total cellular RNA was extracted with the RNAXEL kit (Eurobio, Les Ullis, France) (Chomczinski and Sacchi, 1987). RNA was quantitated by absorbance at 260 nm. Approximately 1 μg of total RNA was used for reverse transcription (RT) reaction with random primers. We then amplified the various tubulin isotype genes and $\beta_2$ microglobulin ($\beta_2$M), the internal standard, with *Taq* polymerase (Appligen, Illkirch, France).

Primer sequences were:

$\beta_2$M (sense): 5′ CCGACATTGAAGTTGACTTAC 3′
$\beta_2$M (antisense): 5′ ATCTTCAAACCTCCATGATG 3′
M40 (class I) (sense): 5′ CCATACATACCTTGAGGCGA 3′
M40 (class I) (antisense): 5′ GCCAAAAGGACCTGAGCGAA 3′
$\beta_9$ (class II) (sense): 5′ CGCATCTCCGAGCAGTTCAC 3′
$\beta_9$ (class II) (antisense): 5′ TCGCCCTCCTCCTCCTCGA 3′
$\beta_4$ (class III) (sense): 5′ ATGAGGGAAATCGTG 3′
$\beta_4$ (class III) (antisense): 5′ AAAGGCCCCTGAGCGGACACT 3′
$\beta_5$ (class IVa) (sense): 5′ TCTCCGCCGCATCTTCCA 3′
$\beta_5$ (class IVa) (antisense): 5′ TCTGGGGACATAATTTCCTC 3′
$\beta_2$ (class IVb) (sense): 5′ GACGGTCGGTTGTAGCACTCTG 3′
$\beta_2$ (class IVb) (antisense): 5′ CAGCACCACTCTGACCGAAAA 3′.

Class I, III, IVa and IVb β-tubulin primers were designed to span introns. In the case of the class II β-tubulin isotype, sequence was obtained from the EMBL GeneBank (accession number X7 and 9353), and using the peptide sequence previously reported by Cowan et al (1986). In separate experiments the presence of tubulin pseudogenes was analysed by performing PCR directly on the RNA, and by digesting RNA with DNAase before cDNA synthesis.

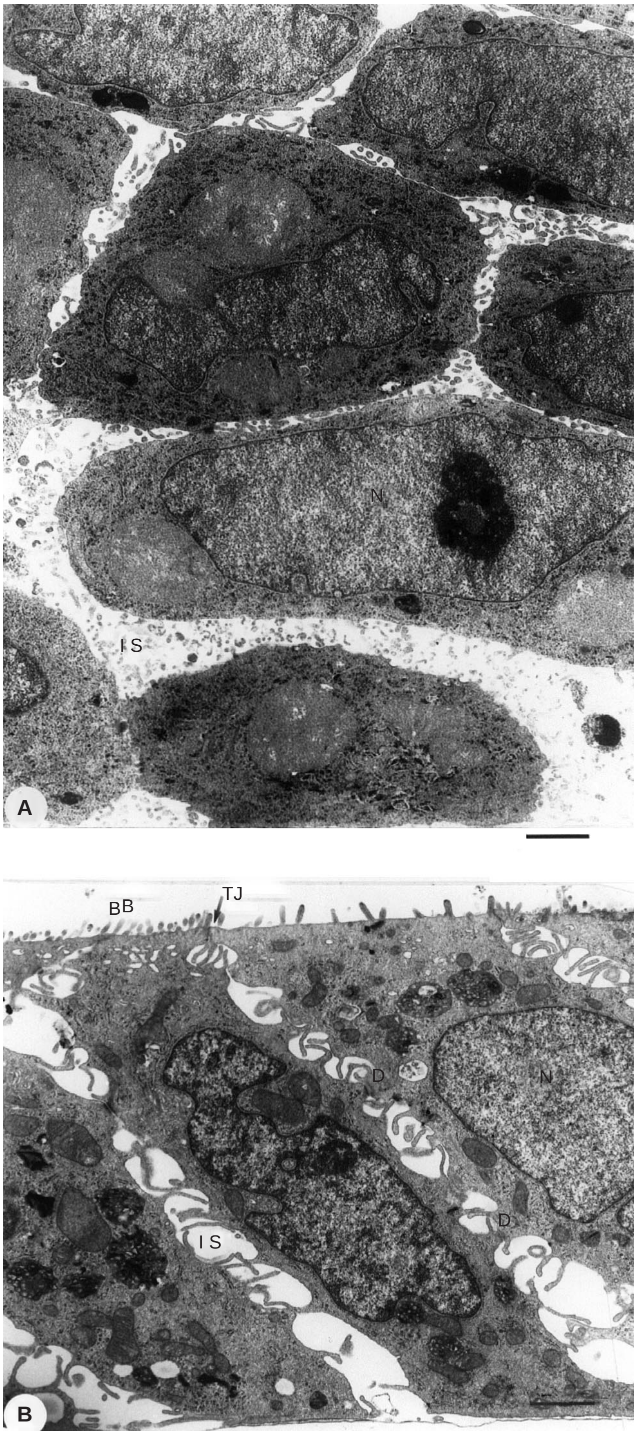

**Figure 1** Transmission electron micrographs of HT29-D4 cells. HT29-D4 Glu cells (**A**) grown in *Glu* medium are undifferentiated and form a non-polarized cell multilayer. HT29-D4 *Gal* cells (**B**) grown in *Gal* medium for 30 days are organized into a polarized monolayer with apical brush border (BB) and tight junction (TJ). D: desmosome, IS: intercellular space, N: nucleus. Bar = 3.3 μm

*© 1999 Cancer Research Campaign*

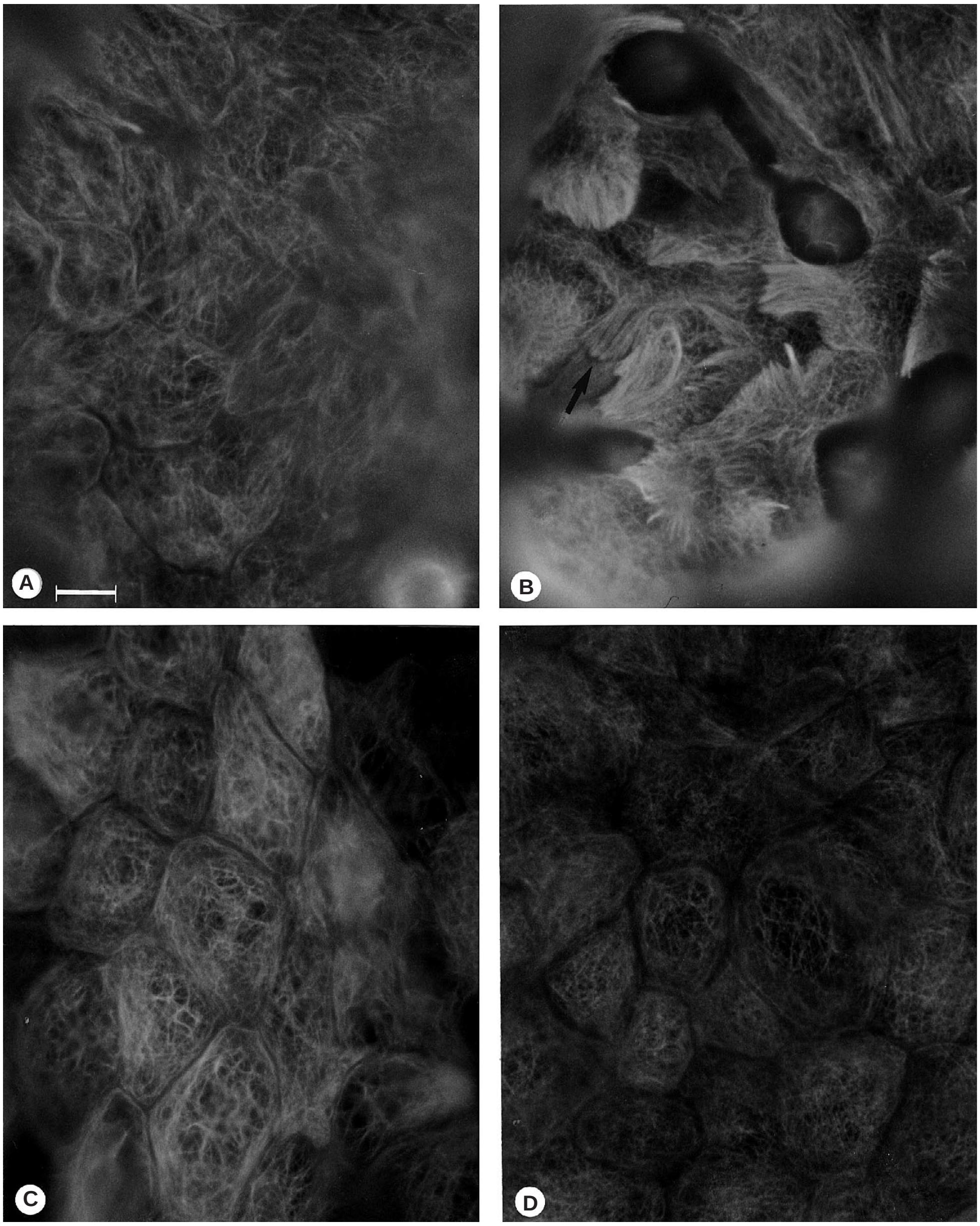

**Figure 2** Indirect immunofluorescence staining of microtubular network with α-tubulin mAb. Undifferentiated HT29-D4 *Glu* (**A**) and differentiated HT29-D4 *Gal* (**C**) control cells; HT29-D4 *Glu* cells treated with paclitaxel 50 nM for 6 h (**B**) and HT29-D4 *Gal* cells treated with paclitaxel 1 μM for 24 h (**D**). Bundles (arrow) were detected only in paclitaxel-treated undifferentiated HT29-D4 *Glu* cells. Bar = 10 μm

PCR was carried out in a Perkin-Elmer system 2400 thermocycler. The reaction conditions included an initial cycle of denaturation at 94°C for 2 min, followed by 25–35 cycles of denaturation at 94°C for 10 s, annealing at 50°C (class I, II, IVa and IVb) or 55°C (class III) for 30 s, and extension at 72°C for 30 s. The amplified products were separated by electrophoresis on a 2% agarose gel. The DNA bands were visualized by ethidium bromide staining, and the image was digitized by an Appligen Imager. Gene expression was normalized to β2M transcript; this was noted REL for relative expression level: REL = densitometric value of studied gene/densitometric value of β2M.

### RESULTS

### Cell differentiation

Electron microscopy showed that HT29-D4 *Glu*, i.e. undifferentiated cells, formed a non-polarized cell multilayer (Figure 1A) whereas HT29-D4 *Gal* cells showed morphological changes characteristic of the differentiated phenotype such as cell monolayer with tight junctions and an apical brush border (Figure 1B). CEA was faintly expressed at the surface of HT29-D4 *Glu* cells; for HT29-D4 *Gal* cells, it was expressed at the apical side and released

© 1999 Cancer Research Campaign 

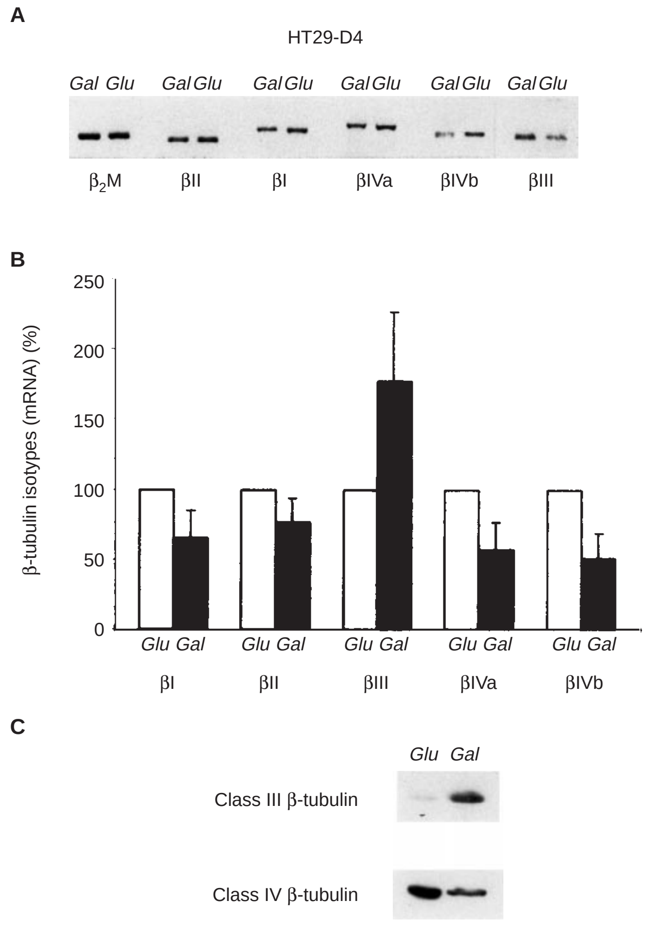

**Figure 3** (**A,B**) β-tubulin isotypes in HT29-D4 *Glu* cells and HT29-D4 *Gal* cells were analysed by RT-PCR. $\beta_2$M (220 pb) was used as an endogenous control. PCR products showed significant increases in βI (288 pb), βII (189 pb), βIVa (271 pb), βIVb (342 pb) transcripts in HT29-D4 *Glu* cells whereas βIII (243 pb) transcripts were lower than those of HT29-D4 *Gal* cells. (**A**) An example of such experiments. (**B**) β-tubulin isotypes in HT29-D4 *Gal* cells were quantified and expressed comparatively to their amount arbitrary evaluated at 100% in HT29-D4 *Glu* cells. (**C**) Western blot analysis of proteins isolated from HT29-D4 *Glu* and HT29-D4 *Gal* cells. Total cell lysates were separated on 10% polyacrylamide gels and stained with class III and class IV β-tubulin mAbs. The images show a marked increase in the class III β-tubulin isotype and a decrease in the class IV-β-tubulin isotype in HT29-D4 *Gal* cells in a concentration dependent manner (data not shown). $\beta_1$ integrin, a polarity marker selectively expressed at the baso-lateral side of differentiated cells, showed no immunofluorescence in HT29-D4 *Gal* cells (data not shown).

### Activity of taxoids according to the cell phenotype

### *Modification of microtubular network*

Paclitaxel and docetaxel induced a concentration- and time-dependent formation of bundles in HT29-D4 *Glu* cells (10–1000 nM, 4–6 h), whereas no bundles were observed in HT29-D4 *Gal* cells even for contact times as long as 96 h (Figure 2).

### *Intracellular drug concentration*

Intracellular paclitaxel and docetaxel concentrations, expressed in picomoles per milligram of protein, were twofold higher in HT29-D4 *Gal* cells than in HT29-D4 *Glu* cells (Table 1).

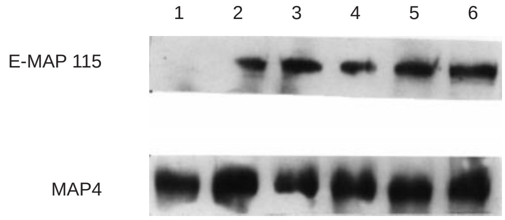

**Figure 4** Western blot analysis of E-MAP115 and MAP4 in HT29-D4 Glu cells (lane 1) and during the course of the differentiation at days 14 (lane 2), 19 (lane 3), 26 (lane 4), 30 (lane 5), 35 (lane 6). Establishment of polarity, assessed by CEA release was apparent from day 19

### Modifications of the microtubular network according to the cell phenotype

### *Cell tubulin content*

Western immunoblots from at least three independent experiments issued from separate cultures are analysed in Table 2. The two phenotypes of HT29-D4 cells contained the same amount of polymerized α-tubulin whatever the polarity status, whereas the total cell tubulin content was significantly higher in undifferentiated HT29-D4 *Glu* cells. Similar results were obtained by quantification of β-tubulin. Total tyrosinated α-tubulin was largely more expressed in HT29-D4 *Glu* cells; on the contrary detyrosinated α-tubulin was twofold higher in microtubules isolated from HT29-D4 *Gal* cells. The amount of acetylated α-tubulin in microtubules was unchanged.

### *Isotypes of β-tubulin*

β-tubulin transcripts were first analysed by RT-PCR. Isotype content was normalized to $\beta_2$M content (Figure 3A). HT29-D4 *Gal* cells displayed much more class III β-tubulin isotype than HT29-D4 *Glu* cells (PCR ratio *Gal*/PCR ratio *Glu* = 1.7 ± 0.50), contrary to class I, II, IVa and IVb β-tubulin isotypes (Figure 3B). By immunoblotting, overexpression of class III β-tubulin isotype in HT29-D4 *Gal* cells was confirmed at the protein level whereas the class IV β-tubulin protein content was lower in HT29-D4 *Gal* cells than in HT29-D4 *Glu* cells (Figure 3C).

### *MAP*

Note that E-MAP-115 was very slightly expressed in HT29-D4 *Glu* cells and significantly increased in the differentiated phenotype (Figure 4). The expression of E-MAP-115 was correlated with the cell polarity evaluated with CEA release. E-MAP-115 was expressed at the beginning of the differentiation process and then it did not vary while polarity was established (from day 19). No variation was detected in the expression of MAP4 by using immunodetection with mAb (Figure 4).

### DISCUSSION

The model HT29-D4 cell line issued from human colon adenocarcima cells cloned by the limiting dilution technique leads to a homogeneous cell population. After 1 month of galactose treatment, cell differentiation was complete, as shown by ultrastructural observations and expression of typical polarity markers.

We have previously shown that the cytotoxicity of anti-microtubule agents on HT29-D4 cells differed markedly according to the differentiation status (Carles et al, 1998). In the present study, we first characterized the action of taxoids at the microtubular

 © 1999 Cancer Research Campaign level on both phenotypes independently of the mitotic process. Microtubular bundles were seen in undifferentiated cells (HT29-D4 *Glu*) after a 4–6 h treatment with paclitaxel or docetaxel. This duration of treatment was too short to induce a mitotic block. In similar conditions, and even for 500-fold higher concentrations of the two drugs and longer treatment, bundle formation was never observed in differentiated cells (HT29-D4 *Gal*). This phenomenon cannot be due to a difference in intracellular drug concentration because it was higher in HT29-D4 *Gal* cells, probably because of a larger membrane area or a modification in its composition. Moreover HT29-D4 does not express the mdr-1 gene (Dimanche-Boitrel et al, 1993). The striking contrast in sensitivity of these two phenotypes to taxoids led us to study the changes in microtubular network according to the differentiation process.

Immunoblots show that microtubular protein amount was identical for both phenotypes whereas the total tubulin was higher in HT29-D4 *Glu* cells. As expected, the analysis of the post-translational modifications showed a twofold higher expression of detyrosinated α-tubulin in microtubules isolated from HT29-D4 *Gal* cells whereas tyrosinated tubulin was expressed more in HT29-D4 *Glu* cells. These results suggest that microtubules in non-polarized HT29-D4 *Glu* cells may be highly dynamic since tyrosinated tubulin dimers are predominant, and that the microtubule turnover is largely decreased during differentiation, may be because the microtubular network is more stable. Acetylation, another post-translational modification, does not seem involved in the differentiation process of HT29-D4 cells. However, it cannot be excluded that acetylation may modify discrete regions along stable microtubules. This modification may be too slight to be quantitatively detected by Western blotting.

Our results indicate that the class I, II, III, IVa and IVb isotypes were expressed in HT29-D4 cells whatever the differentiation phenotype. With the exception of class III β-tubulin isotype, the isotypes were expressed more in HT29-D4 *Glu* cells than in HT29-D4 *Gal* cells but their relative amount did not differ significantly from that of the total tubulin. Since their expression varies according to tubulin level, it appears that these variations are not involved in the polarity status. Interestingly, the class III β-tubulin isotype mRNA was expressed at low level in HT29-D4 *Glu* cells and was increased 1.7-fold in differentiated enterocyte-like cells. The overexpression of class III β-tubulin isotype was confirmed at the protein level. Furthermore, the change in expression of β-III isotype, which is much greater at the protein level than at mRNA level, suggests that microtubules from αβ-III are stable in differentiated cells.

We demonstrate that the increase in class III β-tubulin isotype is linked to the differentiation process in epithelial cells, as reported for differentiated neuronal cells (Gard and Kirshner, 1985; Joshi and Cleveland, 1989). Little is available on the tubulin isotype composition of normal or tumour cells; moreover, functional differences between isotypes that may be generalized to a variety of cell models have not been identified (Luduena, 1993). Furthermore, β-tubulin isotypes may undergo post-translational modifications like glutamylation (Alexander et al, 1991; Rudiger et al, 1992; Mary et al, 1994) or phosphorylation in the carboxy terminal domain that is functionally important for the interaction with MAPs.

Other factors such as MAPs are involved in the events of microtubule stabilization (Matus, 1988; Hirokawa, 1994). One could expect epithelial cell-specific MAPs to be involved in microtubule stabilization during epithelial cell polarization. MAP4 is the predominant MAP of non-neuronal cells in mammalian proliferating and differentiating cells (Bulinski and Borisy, 1979). E-MAP-115, predominantly expressed in cells of epithelial origin, may be another candidate for a protein regulating microtubule stability and reorganization during cell polarization, as shown by the strong increase in labelling in Caco-2 polarized cells (Masson and Kreis, 1993). We quantified the expression of MAP4 and E-MAP-115 during the polarization process. No variation in the expression of MAP4 was observed during HT29-D4 cell differentiation, suggesting that MAP4 does not play a significant role in this process. This data confirms that removal of MAP4 from microtubules with a blocking Ab in vivo does not modify the phenotype at the cellular level (Wang et al, 1996). E-MAP-115 was expressed at very low levels in undifferentiated cells, but was largely increased after the beginning of the polarity process, before CEA was released and the cell monolayer was established. Consequently, E-MAP-115 is involved in the initial events of polarization in HT29-D4 cells, as previously reported for the colon Caco-2 cell line (Masson and Kreis, 1995).

Altogether, our results show two major differences in the microtubular network of HT29-D4 cells according to the cell phenotype: overexpression of class III β-tubulin isotype and E-MAP-115 in microtubules in differentiated cells. Each of these changes may contribute to a higher stability of microtubular network in differentiated cells, beside detyrosination of α-tubulin. Indeed, E-MAP-115 is involved in stabilizing microtubules in cells as shown in fibroblasts transfected with E-MAP-115 cDNA. Moreover, β-tubulin isotype composition can regulate microtubule dynamics, as demonstrated in vitro with αβ-III dimers. We also suggest that the stabilizing function of E-MAP-115 is strengthened by the overexpression of class III β-tubulin isotype. Consequently, microtubule dynamics may be reduced in differentiated cells, thereby inhibiting the activity of anti-microtubule agents independently of the mitotic process. Microtubules from α-βIII dimers display a mean shortening rate lower than that of unfractionated microtubules and they are less sensitive to paclitaxel (Derry et al, 1997). Moreover, an increase in the class III β-tubulin isotype has been involved in tumour cells resistant to paclitaxel (Kavallaris et al, 1997; Ranganathan et al, 1998). Our data are of particular interest in cancer therapy because of the high frequency of human tumours of epithelial origin and because the differentiation status is one of the major prognostic factors. Moreover, the present report indicates that the microtubule network may influence the activity of anti-microtubule agents. The chemosensitivity of tumours varies according to the phenotype of cell differentiation; it is noteworthy that proliferating status is often associated with a higher sensitivity to anticancer drugs. Epithelial tumours are often heterogeneous regarding to their differentiation phenotypes. Our results suggest that the most differentiated cells, via alteration of microtubule network, might promote the emergence of clinical resistance against anti-microtubule agents, when used at conventional doses. Thus, the biochemical composition of microtubules should be considered one of the criteria involved in the response of tumour cells to chemotherapy with these drugs. Furthermore, since HT29-D4 cells display a selective increase in the class III β-tubulin isotype in the differentiated phenotype, this model will help to further investigate the functional role of this isotype, which has not yet been clearly defined.

### ACKNOWLEDGEMENTS

Dr Thomas Kreis, who provided the *Glu* tubulin antibody, was tragically killed in the Swissair crash in 1998. This was a great loss for the scientific community and Dr Th Kreis will be sorely missed by all of us. We thank Danièle Masson for E-MAP-115 antibody and for critical reading of our manuscript, Jeanette Chloë Bulinski and Anthony Frankfurter for MAP4 and class III β-tubulin isotype antibodies, Monique Roccabianca for transmission electron microscopy and Charles Prevost for flow cytometric measurements.

### REFERENCES

Alexander JE, Hunt DF, Lee MK, Shabanowitz J, Michel H, Berlin SC, Macdonald RJ, Rebhun LI and Franckfurter A (1991) Characterization of posttranslational modifications in neuron-specific class III β-tubulin by mass spectrometry. *Proc Natl Acad Sci USA* **88**: 4685–4689

Arruda M, Cocchiaro CA, Nelson CM, Grinnel CM, Janssen B, Haupt A and Barlozzari T (1995) LU103793 (NSC D-669356): a synthetic peptide that interacts with microtubules and inhibits mitosis. *Cancer Res* **55**: 3085–3092

Baas PW and Black MB (1990) Individual microtubules in the axon consist of domains that differ in both composition and stability. *J Cell Biol* **111**: 495–509

Bulinski JC and Borisy GG (1979) Self-assembly of Hela tubulin and the identification of Hela microtubule proteins. *Proc Natl Acad Sci USA* **76**: 293–297

Bulinski JC and Gundersen GG (1991) Stabilization and post-translational modification of microtubules during cellular morphogenesis. *BioEssays* **13**: 285–293

Carles G, Braguer D, Sabeur G and Briand C (1998) Effect of combining anti-microtubule agents at low doses on differentiated and undifferentiated human colon cancer cell lines. *Anticancer Drugs* **9**: 209–221.

Cassimeris L (1993) Regulation of microtubule dynamic instability in living cells. *Cell Motil Cytoskeleton* **26**: 275–281.

Chomczynski P and Sacchi N (1987) Single-step method of RNA isolation by acid guanidinium thicyanate–phenol–chloroform extraction. *Anal Biochem* **162**: 156–159

Cowan NJ, Lewis SA, Sarkar S and Gu W (1986) Functional versatility of mammalian β-tubulin isotypes. In: *The Cytoskeleton in Cell Differentiation and Development*, Maccioni RB and Arechaga J (eds), pp. 157–166. IRL Press; Oxford

Derry WB, Wilson L, Khan IA, Luduena RF and Jordan MA (1997) Taxol differentially modulates the dynamics of microtubule assembled from unfractionated and purified β-tubulin isotypes. *Biochemistry* **6**: 3554–3562

Dieras V, Fumoleau P, Bourgeois H, Misset JL, Azli N and Pouillart T (1996) Taxoids in combination chemotherapy for metastatic breast cancer. *Anticancer Drugs* **7**: 47–52.

Dimanche-Boitrel MT, Garrido C and Chauffert B (1993) Kinetic resistance to anticancer agents. *Cytotechnology* **12**: 5596–5602

Falconer MM, Echeverri CJ and Brown DL (1992) Differential sorting of β-tubulin isotypes into colchicine-stable microtubules during neuronal and muscle differentiation of embryonal carcinoma cells. *Cell Motil Cytoskeleton* **21**: 313–325.

Fantini J, Abadie B, Tirard A, Remy L, Ripert JP, El Battari A and Marvaldi J (1986) Spontaneous and induced dome formation by two clonal cell populations derived from a human adenocarcinoma cell line, HT29. *J Cell Sci* **83**: 235–249

Fantini J, Rognoni JB, Culouscou JM, Pommier G, Marvaldi J and Tirard A (1989) Induction of polarized apical expression and vectorial release of carcinoembryonic antigen (CEA) during the process of differentiation of HT29-D4 cells. *J Cell Physiol* **141**: 126–134

Garcia P, Braguer D, Carles G, El Khiari S, Barra Y, De Ines C, Barasoain I and Briand C (1994) Comparative effects of taxol and taxotere on two different human carcinoma cell lines. *Cancer Chemother Pharmacol* **34**: 335–343

Gard DL and Kirshner MW (1985) A polymer-dependent increase in phosphorylation of β-tubulin accompanies differentiation of mouse neuroblastoma cell line. *J Cell Biol* **100**: 764–774

Gurland G and Gundersen GG (1995) Stable detyrosinated microtubules function to localize vimentin intermediate filaments in fibroblasts. *J Cell Biol* **131**: 1275–1290

Hirokawa N (1994) Microtubule organization and dynamics dependent on microtubule-associated proteins. *Curr Opin Cell Biol* **6**: 74–81.

Horwitz SB, Cohen D, Rao S, Ringel I, Shen HJ and Yang CP (1993) Taxol: mechanisms of action and resistance. *J Natl Cancer Inst Monogr* **15**: 55–61

Jordan MA and Wilson L (1998) Microtubules and actin filaments: dynamic targets for cancer chemotherapy. *Curr Opin Cell Biol* **10**: 123–130

Joschi HC and Cleveland DW (1989) Differential utilization of β-tubulin isotypes in differentiating neurites. *J Cell Biol* **109**: 663–673

Kavallaris M, Kuo DY, Burkhart CA, Regl DL, Norris MD, Haber M and Horwitz SB (1997) Taxol-resistant epithelial ovarian tumors are associated with altered expression of specific β-tubulin isotypes. *Cancer Res* **100**: 1282–1293

Luduena RF (1993) Are tubulin isotypes functionally significant? *Mol Biol Cell* **4**: 445–457

Manfredi JJ and Horwitz SB (1984) Taxol: an antimitotic agent with a new mechanism of action. *Pharmacol Ther* **25**: 83–125

Mary J, Redeker V, Le Caer JP, Prome JG and Rossier J (1994) Class I and $IV_a$ β-tubulin isotypes expressed in adult mouse brain are glutamylated. *FEBS Lett* **353**: 89–94

Masson D and Kreis TE (1993) Identification and molecular characterization of E-MAP-115, a novel microtubule-associated protein predominantly expressed in epithelial cells. *J Cell Biol* **123**: 357–371.

Masson D and Kreis TE (1995) Binding of E-MAP-115 to microtubules is regulated by cell cycle-dependent phosphorylation. *J Cell Biol* **131**: 1015–1024

Matus A (1988) Microtubule associated proteins: their potential role in determining neuronal morphology. *Annu Rev Neurosci* **11**: 29–44

McGuire WP, Hoskins WJ, Brady MF, Kucera PR, Partridge EE, Look KY, Clarke-Pearson DL and Davidson M (1996) Cyclophosphamide and cisplatin compared with paclitaxel and cisplatin in patients with stage III and stage IV ovarian cancer. *N Engl J Med* **334**: 1–6

Ranganathan S, Benetatos CA, Colarusso PJ, Dexter DW and Hudes GR (1998) Altered β-tubulin isotype expression in paclitaxel-resistant human prostate carcinoma cells. *Br J Cancer* **77**: 562–566

Rognoni JB, Pichard V, Honore S, Rigot V, Lehmann M, Roccabianca M, Carles G, Luis J, Marvaldi J and Briand C (1998) Convergent effects of growth factors, hormones, and fibronectin are necessary for the enterocyte differentiation of a colon adenocarcinoma cell line (HT29-D4). *Differentiation* **63**: 305–317

Rowinsky EK, Onetto N and Canetta RA (1992) Taxol: the first of the taxanes, an important new class of antitumor agents. *Semin Oncol* **19**: 646–662

Rudiger M, Plessman U, Kloppel KD, Wehland J and Weber K (1992) Class II tubulin, the major brain β-tubulin isotype is polyglutamylated on glutamic acid residue 435. *FEBS Lett* **308**: 101–105

Schibler MJ and Cabral F (1986) Taxol-dependent mutants of chinese hamster ovary cells with alterations of α- and β-tubulin. *J Cell Biol* **102**: 1522–1531

Shelden E and Wadsworth P (1993) Observation and quantification of individual microtubule behavior in vivo: microtubule dynamics are cell-type specific. *J Cell Biol* **120**: 935–945

Simons K and Wandinger-Ness A (1990) Polarized sorting in epithelia. *Cell* **62**: 207–210

Wadsworth P and Bottaro DP (1996) Microtubule dynamic turnover is suppressed during polarization and stimulated in hepatocyte growth factor scattered Madin-Darby Canine Kidney epithelial cells. *Cell Motil Cytoskeleton* **35**: 225–236

Wang XM, Peloquin JG, Zhai Y, Bulinski JC and Borisy GG (1996) Removal of MAP4 from microtubules in vivo produces no observable phenotype at the cellular level. *J Cell Biol* **132**: 345–357

Wilson L and Jordan MA (1995) Microtubule dynamics: taking aim at a moving target. *Chem Biol* **2**: 569–573

 *© 1999 Cancer Research Campaign*
